# The prognostic value of long noncoding RNA HOTTIP on clinical outcomes in breast cancer

**DOI:** 10.18632/oncotarget.14304

**Published:** 2016-12-27

**Authors:** Yinlong Yang, Jinxian Qian, Youqun Xiang, Yizuo Chen, Jinmiao Qu

**Affiliations:** ^1^ Department of Breast Surgery, Fudan University Shanghai Cancer Center, Department of Oncology, Shanghai Medical College, Fudan University, Shanghai 200000, People's Republic of China; ^2^ Department of Surgical Oncology, The First Affiliated Hospital of Wenzhou Medical University, Wenzhou 325000, Zhejiang, People's Republic of China

**Keywords:** long non-coding RNA, HOTTIP, prognosis, breast cancer

## Abstract

Although a few studies have assessed the prognostic value of long noncoding RNA HOTTIP in patients with malignant tumors, the relationship between HOTTIP and clinical outcome of breast cancer remains elusive. The aim of this study is to explore the prognostic significance of HOTTIP in breast cancer patients. A meta-analysis was performed to involve the eligible studies to investigate the association of HOTTIP expression level with outcome in cancer patients. Pooled hazard ratios (HRs) and 95% confidence interval (CI) of HOTTIP for cancer survival were calculated. Five relevant articles involving 460 patients with various solid carcinomas were included in this meta-analysis. For overall survival, high HOTTIP expression could significantly predict worse outcome with the pooled HR of 2.29 (95 % CI 1.72–3.03, P < 0.00001). Furthermore, Gene Expression Omnibus was performed to evaluate the association of HOTTIP expression with the prognosis in breast cancer patients. It was also found an indication that high HOTTIP expression was associated with worse survival in breast cancer patients by microarray analysis (GSE20711, GSE16446 and GSE9195). Finally, association between HOTTIP levels and clinicopathological factors and prognosis was also analyzed in an independent validation cohort including 100 breast cancer cases. HOTTIP expression was correlated with tumor size (P=0.025), lymph node status (P=0.009) and TNM stage (P=0.0001) in the breast cancer validation cohort. The Kaplan-Meier survival curves indicated that breast cancer patients with high HOTTIP expression had worse overall survival (P=0.0139) and disease-free survival (P=0.0003). Multivariate survival analysis based on the Cox proportional hazards model showed that HOTTP is considered as an independent prognostic factor in breast cancer patients. Together, our combined results suggest that high HOTTIP expression may be serving as an unfavorable prognosis predictor for breast cancer patients.

## INTRODUCTION

Breast cancer (BC) is the most common cancer in women with 246,660 newly estimated diagnosed cases and40,450 estimated deaths in the United States in 2016 [1]. Remarkable progresses have been made during recent decades. However, BC is a highly heterogeneous disease, which is diverse in its responsiveness to treatment [2, 3]. Thus, there has been increasing interest on identifying novel prognostic biomarkers to predict its biological behavior. New biomarkers can also improve treatment design and contribute to the development of new therapeutic targets.

Long noncoding RNAs (lncRNAs) are defined as RNA molecules with more than 200 nucleotides in length not translated into protein. Once considered as transcriptional noise, lncRNAs have now been shown to be involved in genome packaging, chromatin organization, dosage compensation, genomic imprinting and gene regulation [4]. Recently, emerging evidences have demonstrated the characterization of lncRNAs as a critical component in cancer biology [5, 6]. Abnormally expressed lncRNAs have either oncogenic or tumor suppressor functions during cancer initiation, development and progression [7]. Given that many lncRNAs are expressed in the way of tissue and cancer type restriction, specific lncRNAs are now likely to be translated into clinical applications for diagnosis, prognosis or predicting the response of treatment [8, 9]. The HOXA transcript at the distal tip (HOTTIP) lncRNA, located at the 5′end of the HOXA cluster, was recently functionally characterized [10]. Increased HOTTIP expression has been reported in tongue squamous cell carcinoma [11], lung cancer [12], pancreatic cancer [13], hepatocellular carcinoma [14], osteosarcoma [15], gastric cancer [16], glioma [17] and prostate cancer [18]. In these tumors, HOTTIP may serve as a potential oncogene and be an adverse prognostic factor in patients. However, little is known about the significance of HOTTIP expression and BC prognosis to date.

In this study, we performed a meta-analysis to investigate the relationship between HOTTIP expression and the survival in patients with various cancers. Additionally, Gene Expression Omnibus (GEO) dataset were selected to disclose the association between the expressions of HOTTIP and BC prognosis. Furthermore, we conducted a validation set to verify the prognostic significance of HOTTIP in BC patients.

## RESULTS

### Meta-analysis and microarray analysis

**Figure 1:**
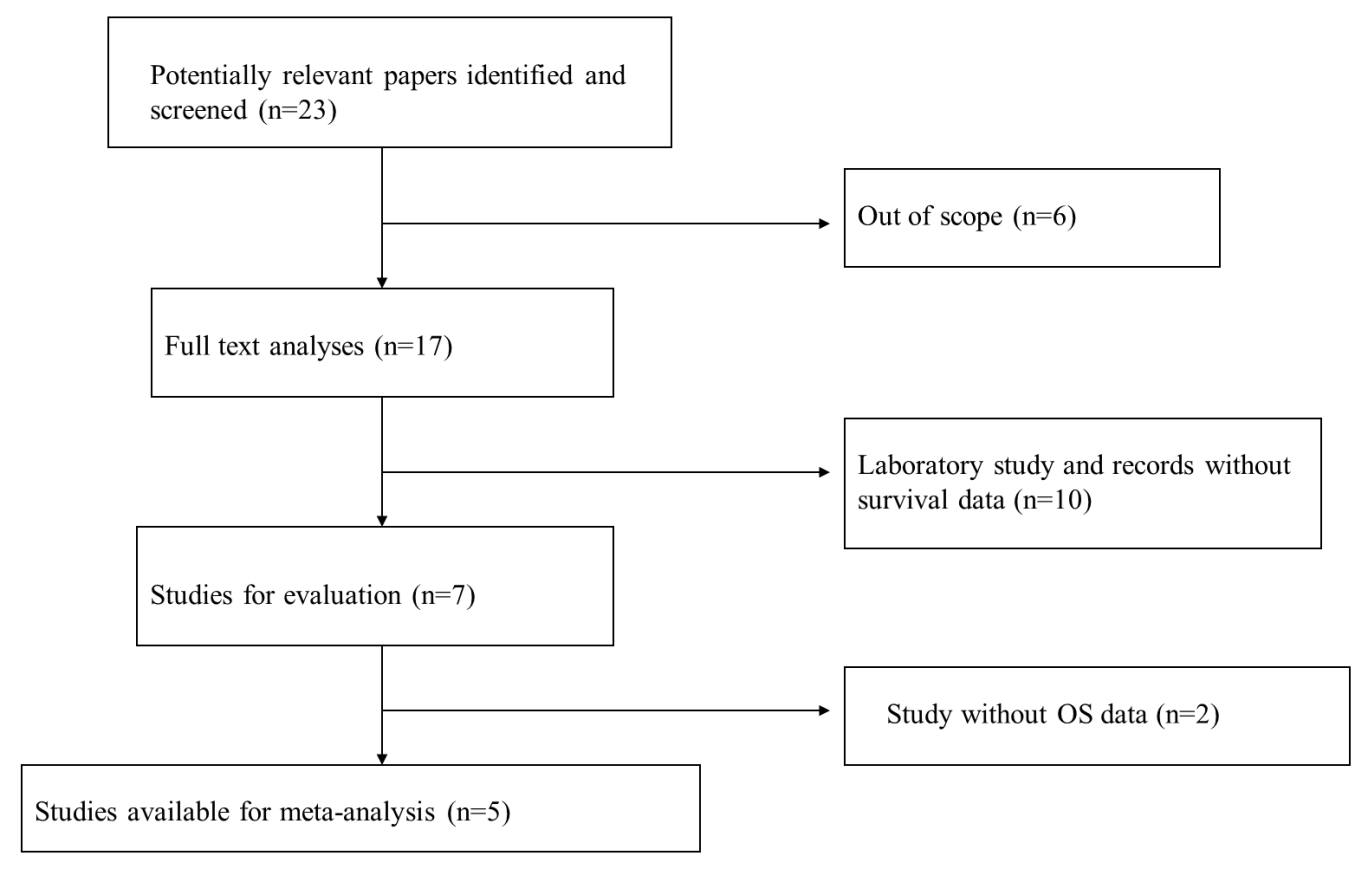
Flow diagram of the identification and selection of studies

**Figure 2:**
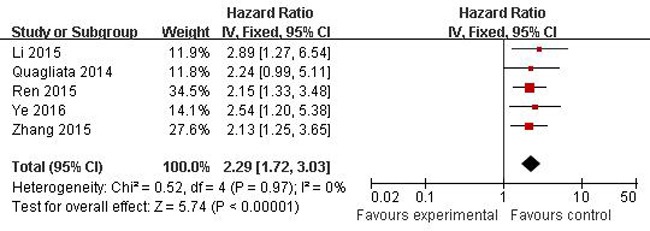
Forest plots of studies evaluating hazard ratios of overall survival comparing high and low HOTTIP expression among patients with solid cancer

**Figure 3:**
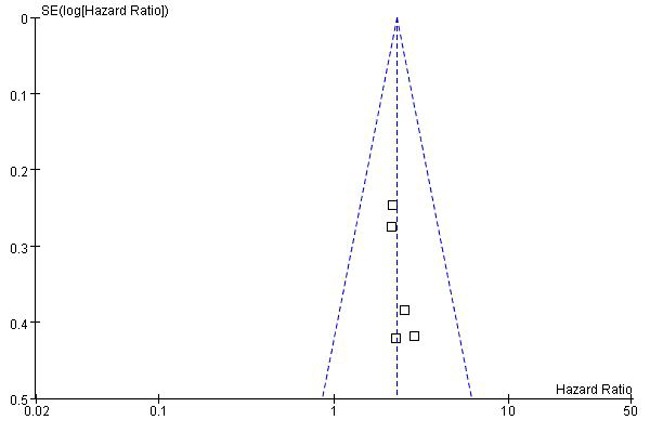
Funnel plot for identifying publication bias in the association between HOTTIP expression and overall survival of patients with solid tumors

A total of 23 studies using the above search strategy were retrieved. A flow diagram of the identification and selection of studies is shown in Figure 1. After evaluation, five studies including 460 patients were finally enrolled for further analysis [11, 14–16, 21]. The sample size ranged from 52 to 156 patients. For all studies, expression of HOTTIP was assessed by RT-PCR. These studies were all retrospective in design. Four studies evaluated patients from China, one evaluated patients from Switzerland. The types of cancers in these studies included hepatocellular cancer, tongue squamous cell carcinoma, colorectal cancer, osteosarcoma and gastric cancer. Table 1 presented the detailed parameters of the included studies. In the fixed effects model, meta-analysis of 5 studies on the prognostic value of HOTTIP expression indicated that higher expression level of HOTTIP significantly predicted worse overall survival (OS) in various solid carcinomas, with the pooled HR of 2.29 (95 % CI 1.72–3.03, P < 0.00001; Figure 2). Finally, funnel plot and Egger's test were used to evaluate the publication bias of the included studies. The shape of the funnel plot did not reveal any evidence of obvious asymmetry (Figure 3).

**Table 1:** Characteristics of the all included studies in the meta-analysis

Study	Year	Country	Cancer type	Number	Sample	Cut-off	Method	Outcome	Follow-up(months)
Quagliata	2014	Switzerland	Hepatocellular cancer	52	Tissue	ROC	qRT-PCR	OS	≥60
Zhang	2014	China	Tongue squamous cell carcinoma	86	Tissue	Median	qRT-PCR	OS	38(range,23–60)
Ren	2015	China	Colorectal cancer	156	Tissue	Median	qRT-PCR	OS	46(range,33-65)
Li	2015	China	Osteosarcoma	68	Tissue	Median	qRT-PCR	OS	≥60
Ye	2016	China	Gastric cancer	98	Tissue	Median	qRT-PCR	OS	≥60

ROC, receiver operating characteristic; qRT-PCR, quantitative real-time polymerase chain reaction; OS, overall survival.

**Figure 4:**
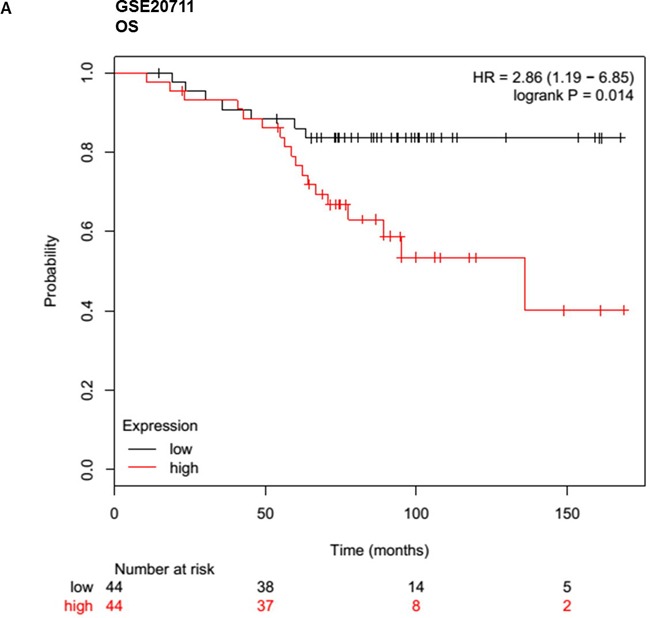
Up-regulated HOTTIP was associated with poor prognosis in breast cancer Overall survival (OS) and relapse free survival (RFS) analysis of patients with low and high HOTTIP expression using a Kaplan-Meier curve in the GSE20711 **A**. GSE16446 **B**. and GSE9195. **C**. datasets. HR: hazard ratio.

Given that meta-analysis identified that aberrant overexpression of HOTTIP is associated with unfavorable survival in patients with various solid cancer. There is no available report on the relationship between HOTTIP and BC. Hence, we performed Gene Expression Omnibus (GEO) dataset parameters to disclose the association between the expression of HOTTIP and prognosis of BC. Three microarray datasets (GSE20711, GSE16446, GSE9195) which included BC patients were collected from the GEO. It demonstrated that BC patients with high HOTTIP expression had worse OS (GSE20711, GSE16446) and relapse free survival (GSE9195) (Figure 4).

### Correlations between HOTTIP expression and clinical characteristics in BC patients

To further validate whether the *HOTTIP* could predict the prognosis in BC patients, we conducted an independent validation set including 100 BC cases by the method of qRT-PCR. BC tissue samples were classified into low-expression group (n=50) and high-expression group (n=50), according to the median expression level of all BC samples. The association between HOTTIP expression levels and clinicopathological characteristics in patients with BC was showed in Table 2. Overall, high HOTTIP expression levels were positively correlated with tumor size, lymph node status and TNM stage (P=0.025, 0.009, 0.0001, respectively). However, HOTTIP expression in BC was not associated with other parameters such as age (p=0.161), menopausal status (p=0.728), grade (p=0.685), ER status (p=0.161), PR status (p=0.422), Her-2 status (p=0.135) (Table 2). These data imply that up-regulated HOTTIP correlates with BC progression.

**Table 2:** Correlation between HOTTIP expression and clinicopathological features in patients with breast cancer

Variables	HOTTIP expression		P value
	Low(n=50)	High(n=50)	
Age			0.161
≦50	23	30	
>50	27	20	
Menopausal status			0.728
Premenopausal	25	32	
Postmenopausal	25	28	
Tumor size			0.025*
≦2cm	26	15	
>2cm	24	35	
Lymph node status			0.009*
Negative	31	18	
Positive	19	32	
Grade			0.685
I-II	20	22	
III	30	28	
ER status			0.161
Negative	21	28	
Positive	29	22	
PR			0.422
Negative	25	29	
Positive	25	21	
HER-2/neu status			0.135
Negative	30	25	
Positive	20	25	
Clinical stage			0.0001*
I-II	42	24	
III	8	26	

### Association between HOTTIP expression and BC patient survival

**Figure 5:**
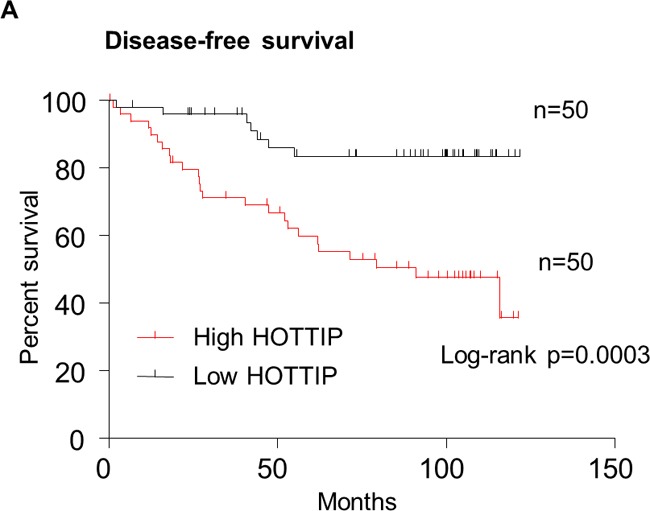
Kaplan–Meier survival curves were stratified by HOTTIP expression level in breast cancer patients Patients in high HOTTIP group showed decreased disease-free survival (DFS, **A**) and overall survival (OS, **B**), as compared with low HOTTIP group, p = 0.0003 and p = 0.0139, respectively. The p value was calculated using the log-rank test.

In order to assess the prognostic value of HOTTIP expression for BC, we analyzed the prognostic value of HOTTIP expression levels in 100 BC patients using Kaplan–Meier analysis and the log-rank test. As shown in Figure 5, patients with high HOTTIP expression had worse disease-free survival (DFS) than those with low HOTTIP expression (P = 0.0003; Figure 5A). More importantly, the high HOTTIP expression group had shorter OS than the low expression group (P = 0.0139; Figure 5B). Moreover, univariate analysis of OS and DFS revealed that HOTTIP overexpression was an unfavorable prognostic factor in BC patients (OS, P=0.002; DFS, P<0.001; Table 3, 4), regardless of other clinicopathological features, such as tumor size, lymph node metastasis, TNM stage, Her-2 status. Finally, Multivariate survival analysis based on the Cox proportional hazards model showed that high HOTTIP expression had an HR of 7.121 for the OS (95% CI=1.176-43.109, P=0.033) and an HR of 4.083 for the DFS (95% CI=1.133–14.713, P=0.03). By comparison, TNM stage had an HR of 2.729 (95% CI 1.103-6.755, P=0.030) for the OS and an HR of 2.555 for the DFS (95% CI 1.219-5.353, P=0.013) (Table 3, 4). Thus, HOTTIP expression was an independent prognostic indicator for survival in BC patients.

**Table 3:** Univariate and multivariate Cox regression analyses of overall survival in breast cancer patients

Variables	Univariate analysis		Multivariate analysis	
	HR(95%CI)	P value	HR(95%CI)	P value
Age(years), ≦50 versus >50	1.245(0.446-3.502)	0.671		
Menopausal status, Premenopausal versus Postmenopausal	1.462(0.520-4.112)	0.472		
Tumor size(cm), ≦2 versus >2	2.411(0.856-6.791)	0.096		
Lymph node status, negative versus positive	3.671(1.196-11.262)	0.023	0.821(0.147-4.587)	0.822
Grade I-II versus III	1.973(0.674-5.770)	0.215		
ER status, negative versus positive	0.247(0.075-0.814)	0.022	0.408(0.052-3.194)	0.393
RR status, negative versus positive	0.190(0.051-0.708)	0.013	0.206(0.024-1.738)	0.147
HER-2/neu status, negative versus positive	5.156(1.754-15.157)	0.003	1.665(0.427-6.941)	0.462
TNM stage, I-II versus III	2.996(1.697-5.290)	0.000	2.729(1.103-6.755)	0.030
HOTTIP expression, low versus high	11.294(2.435-52.397	0.002	7.121(1.176-43.109)	0.033

**Table 4:** Univariate and multivariate Cox regression analyses of disease-free survival in breast cancer patients

Variables	Univariate analysis		Multivariate analysis	
	HR(95%CI)	P value	HR(95%CI)	P value
Age(years), ≦50 versus >50	1.123(0.475-2.660)	0.791		
Menopausal status, Premenopausal versus Postmenopausal	2.003(0.841-4.769)	0.117		
Tumor size(cm), ≦2 versus >2	2.776(1.159-6.649)	0.022	1.849(0.555-6.162)	0.317
Lymph node status, negative versus positive	3.473(1.417-8.515)	0.007	0.772(0.184-3.238)	0.723
Grade I-II versus III	2.837(1.127-7.146)	0.027	1.188(0.328-4.296)	0.793
ER status, negative versus positive	0.579(0.244-1.373)	0.215		
PR status, negative versus positive	0.463(0.190-1.125)	0.089		
HER-2/neu status, negative versus positive	4.943(1.903-12.843)	0.001	2.848(0.802-10.119)	0.106
TNM stage, I-II versus III	3.786(2.095-6.844)	0.000	2.555(1.219-5.353)	0.013
HOTTIP expression, low versus high	9.750(3.319-28.645)	0.000	4.083(1.133-14.713)	0.03

## DISCUSSION

As far as it is concerned, our study is the first report data for the HOTTIP expression and prognosis in patients with BC. In this two-phase study, we systematically investigate the relationship between HOTTIP expression and the survival in patients with various cancers by a meta-analysis. Then, we analyzed the association between the HOTTIP expression and prognosis of BC in three GEO cohorts with BC patients. In the second phase, we evaluated the clinical significance of HOTTIP as a prognostic biomarker for BC patients by using a validation study. We found that HOTTIP expression is associated with the clinicopathological features and prognosis of BC. Thus, HOTTIP can function as a biomarker for malignancy and monitoring prognosis in BC clinically.

Emerging evidence suggests that lncRNAs constitute critical part of tumor biology. Abnormal expression of lncRNAs in cancer marks the spectrum of cancer progression and may serve as an predictor for prognosis in patient s with cancer [9, 22, 23]. Rinn et al. have identified 231 non-coding RNAs associated with human HOX gene loci and these RNAs are spatially expressed and sequence-specific [6]. As the first HOX-associated InRNA that was characterized, HOTAIR was initially identified as a scaffold RNA which was associated with the chromatin-modifying PRC2 complex and the H3K27me3 histone mark, the latter of which is related to gene suppression. Subsequent studies showed that HOTAIR directly interacted with both the PRC2 and LSD1/REST/CoREST repressor complexes [6, 7, 24]. HOTAIR overexpression has been demonstrated in primary breast tumors and metastases. Its overexpression has been shown to alter gene expression pattern of breast epithelial cells to a patter similar to embryonic and neonatal fibroblasts, whereas its down-regulation has changed pattern of H3K27 methylation and decreased invasiveness [7]. Another study has shown that its expression may be an independent biomarker for the prediction of metastasis risk in ER positive BC patients [25]. In addition, its transcription has been shown to be induced by estrogen whereas inhibited by tamoxifen [26]. A recent study indicated its significant overexpression in the HER2-enriched subtype BC [27]. The oncogene functions and unfavorable prognostic significance of HOTAIR have been reported in multiple cancers including BC, whereas the expression and functions of other HOX-associated lncRNAs in cancer cell lines have not been extensively studied.

Recently, HOTTIP has drawn increasing attention among cancer-related lncRNAs, which have been demonstrated to regulate genes by various mechanisms including epigenetic modifications, lncRNA-miRNA and lncRNA-protein interactions [10, 28, 29]. It has been confirmed that HOTTIP could directly interact with the Trithorax protein WDR5 inducing an open DNA-chromatin configuration to target WDR5/MLL complexes driving histone H3 lysine 4 trimethylation and thus regulating the transcription of 5′end HOXA locus genes [10]. Interestingly, recent studies also found that there is a positive correlation between the expression of HOTTIP and HOX genes in tumors and normal tissue [14, 30–33]. It has demonstrated that HOTTIP was involved in the cancer progression. For example, Tsang et al. reported that knockdown of HOTTIP attenuated hepatocellular carcinoma cell proliferation *in vitro* and markedly abrogated tumorigenicity *in vivo*. Furthermore, knockdown of HOTTIP also inhibited cancer cells migration and significantly abrogated lung metastasis in mouse xenograft mode [34]. A similar trend was seen in our study. We also found that that the expression of HOTTIP was positively correlated with tumor size, lymph node metastasis and clinical stage in BC. Furthermore, univariate analysis and multivariate analysis indicated that HOTTIP could be an independent prognostic factor and overexpression of HOTTIP was correlated with unfavorable survival in BC patients. Taken together, our data revealed that HOTTIP was implicated in the progress of BC and it might act as a tumor oncogene. To the best of our knowledge, it was the first report about the association of HOTTIP with the progression and prognosis of BC. However, our study still has several limitations. For one thing, it is a retrospective study, conducted on a small sample. For another, *in vitro* and *in vivo* experiments are needed to be conducted to further validate the biological effects of HOTTIP on breast cancer. The underlying mechanisms also remain unknown and deserve further research.

In summary, our findings indicate that HOTTIP expression level has the potential to be an independent unfavorable prognostic indicator for BC patients. The results of this study indicated that HOTTIP may be a novel prognostic marker and a potential new target for BC therapy.

## MATERIALS AND METHODS

### Meta-analysis search strategy

We performed meta-analysis according to the guidelines of the Meta-analysis of Observational Studies in Epidemiology group (MOOSE) [19]. We searched online PubMed, EMBASE and ISI Web of Science from January 1st, 2011 to October1st, 2016 to identify relevant studies. Two sets of key words were used among that process, namely “HOTTIP and cancer”. A manual review of the references of relevant publications was also performed to obtain more studies.

### Inclusion and exclusion criteria

Eligible studies included in this meta-analysis met the following criteria: (I) they studied the patients with any type of cancer; (II) they measured the expression of HOTTIP; and (III) they investigated the association between HOTTIP expression levels and cancer survival outcome. Articles were excluded on the basis of the following criteria: (I) review articles or letters, (II) non-English articles, (III) animal or laboratory studies. (IV) studies of nondichotomous HOTTIP expression levels, (V)absence of key information about survival outcome such as hazard ratio (HR), 95% CI and P value. When a study reporting the same patient cohort was included in several publications, only the most latest or complete study was selected. If any doubt of suitability remained after the abstract was examined, the complete manuscript was obtained.

### Quality assessment

Two authors critically assessed the quality of all the studies included according to a basic standard as follows: I) clear report of study population and origin of country, II) clear definition of study design, III) clear definition of type of carcinoma, IV) clear definition of outcome assessment, V) clear definition of measurement of HOTTIP, and VI) sufficient follow-up time. Otherwise, the study was removed in order to enhance the quality of the meta-analysis.

### Data extraction

Two reviewers independently extracted the required information from all eligible studies to rule out any discrepancy. The following data were extracted: first author, year of publication, country of origin, sample size, tumor type, method of testing HOTTIP and the cut-off, follow-up, HR of HOTTIP for overall survival (OS) as well as corresponding 95 % confidential interval (CI) and P value. Multivariate Cox hazard regression analysis was used in the present analysis. When HR data were not available but appropriate summary statistics or Kaplan-Meier curves were provided, we calculated HR using the described method [20]. Disagreements were resolved by discussion. All the data were subject to consensus.

### Patients and tissue samples

The 100 cases of clinical specimens were collected from the First Affiliated Hospital of Wenzhou Medical University, between June 2006 and June 2009. All the patients analyzed in this study underwent resection of primary BC. All the cases were histopathologically confirmed as invasive ductal adenocarcinoma. All the resected tissue samples were immediately frozen in liquid nitrogen and stored at −80°C until RNA extraction. The clinical and pathological characteristics of each patient were also collected. All patients completed the follow-up, until 30 July 2016. Overall survival (OS) was defined as the interval between the dates of surgery and death. Disease free survival (DFS) was defined as the interval between the dates of surgery and disease recurrence and distant metastasis. All patients gave written informed consent for the collection of biomaterials. Approval for this study was received from the Ethics Committee of the host institution.

### RNA extraction and quantitative real-time PCR analysis

Total RNA was extracted using TRIzol reagent (Invitrogen). After converting total RNA to cDNA in a reverse transcription (RT) reaction, qPCR were used to quantitate the mRNA expression levels. To detect HOTTIP expression, we used the SYBR Green method. The following primers were used in this study: HOTTIP were 5′-GTGGGGCCCAGACCCGC-3′ (forward) and 5′-AATGATAGGGACACATCGGGGAACT-3′ (reverse).

Beta-actin, 5′-TCCTCTCCCAAGTCCACACA-3′ (forward) and 5-GCACGAAGGCTCATCATTCA-3′(reverse). The relative expression of HOTTIP was calculated and normalized using the delta-delta CT (2^−ΔΔCt^) method relative to Beta-actin. Independent experiments were done in triplicate.

### Statistical analysis

All these HRs and 95% CI were calculated following Tierney's method and the logHR and standard error (logHR) were used for aggregation of the survival results. Generally, an observed HR of >1 implied worse survival for the group with HOTTIP overexpression. This impact of high expression of HOTTIP on the survival rate was considered statistically significant if the 95% CI for the overall HR did not overlap in the forest plot. A test of heterogeneity of combined HRs was carried out using Cochran's Q test and Higgins I-squared statistic. Heterogeneity was defined as p<0.10 or I^2^>50%. Pooled HR was calculated using a fixed effect model or random effect model to evaluate the relationship between HOTTIP expression and overall survival. When homogeneity was fine (p>0.10, I^2^<50%), a fixed effect model was used for secondary analysis. If not, a random effect model was used. Publication bias was evaluated using the funnel plot and Egger's test, P>0.05 was considered indicative of a lack of publication bias.

Using the Kaplan–Meier method, OS and DFS curves were made, and the log-rank test was applied for comparison. The significance of between-group differences was estimated using Student's t-test, χ2 test or Wilcoxon test, as appropriate. Variables with a value of P<0.05 in univariate analysis were used in a subsequent multivariate analysis, based on the Cox proportional hazards model. All P values were two-sided, and all analyses were performed using the Stata soft-ware (StataCorp, College Station, TX, USA) and Review Manager (v5.0; Oxford, United Kingdom).
